# Class-Wide Analysis of Frizzled-Dishevelled Interactions Using BRET Biosensors Reveals Functional Differences among Receptor Paralogs

**DOI:** 10.1021/acssensors.4c00806

**Published:** 2024-08-30

**Authors:** Lukas Grätz, Jan H. Voss, Gunnar Schulte

**Affiliations:** Department of Physiology & Pharmacology, Section of Receptor Biology & Signaling, Biomedicum, Karolinska Institutet, S-17165 Stockholm, Sweden

**Keywords:** Frizzled, Dishevelled, WNT, bioluminescence resonance energy transfer, GPCR, class F, transducer

## Abstract

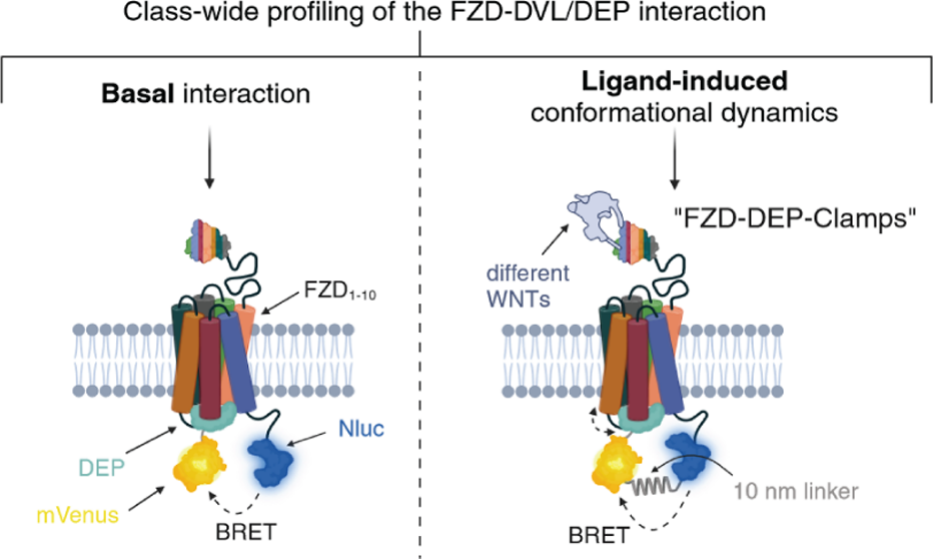

Wingless/Int-1 (WNT) signaling is mediated by WNT binding to 10 Frizzleds (FZD_1–10_), which propagate the signal inside the cell by interacting with different transducers, most prominently the phosphoprotein Dishevelled (DVL). Despite recent progress, questions about WNT/FZD selectivity and paralog-dependent differences in the FZD/DVL interaction remain unanswered. Here, we present a class-wide analysis of the FZD/DVL interaction using the DEP domain of DVL as a proxy in bioluminescence resonance energy transfer (BRET) techniques. Most FZDs engage in a constitutive high-affinity interaction with DEP. Stimulation of unimolecular FZD/DEP BRET sensors with different ligands revealed that most paralogs are dynamic in the FZD/DEP interface, showing distinct profiles in terms of ligand selectivity and signal kinetics. This study underlines mechanistic differences in terms of how allosteric communication between FZDs and their main signal transducer DVL occurs. Moreover, the unimolecular sensors represent the first receptor-focused biosensors to surpass the requirements for high-throughput screening, facilitating FZD-targeted drug discovery.

Morphogens of the Wingless/Int1 (WNT) family play a vital role during embryonic development and govern physiological processes such as regulation of stem cells and tissue homeostasis.^1−3^ Dysregulation of WNT signaling can lead to various pathologies, such as diverse forms of cancer or fibrosis.^4,5^ WNTs are the endogenous ligands of Frizzleds (FZDs), which comprise the class F of the superfamily of G protein-coupled receptors (GPCRs) together with Smoothened (SMO), a key mediator in Hedgehog signaling.^6^ The human genome encodes 19 WNTs and 10 FZDs, whose selectivity for each other is only rudimentarily understood.^7,8^ WNTs bind to the extracellular cysteine-rich domain (CRD) of FZDs and thereby initiate a plethora of complex and intertwined signaling cascades.^6,9^ The complexity of the system is further increased by the contribution of co-receptors, which can coengage WNTs together with FZDs and contribute to signal specification.^10,11^ According to a prevailing model, simultaneous binding of WNTs and the co-receptor low-density lipoprotein receptor-related proteins 5/6 (LRP5/6) leads to the stabilization of the transcriptional regulator β-catenin by inhibition of a multiprotein destruction complex.^12^

FZDs can engage various transducers for signal initiation, most prominently the phosphoprotein Dishevelled (DVL).^13,14^ All vertebrate isoforms of DVL (DVL1–3) contain three structured domains—an N-terminal DIX (Dishevelled and axin) domain, a central Post synaptic density-95/Discs large/Zonula-occludens 1 (PDZ) domain, and a C-terminal Dishevelled, Egl-10, Pleckstrin (DEP) domain—embedded into intrinsically disordered protein stretches.^13,14^ While the DIX domain is involved in basal and WNT-stimulated DVL oligomerization and is indispensable for DVL-dependent signaling,^15,16^ the interaction with FZDs at the cell surface is mostly mediated by the DEP domain.^17,18^ At least in overexpressed systems, the interaction between FZD and DVL/DEP even occurs in the absence of WNTs.^17−20^ Moreover, the importance of a dynamic interplay between FZDs and DVL was suggested over 20 years ago.^21^ Using different BRET (bioluminescence resonance energy transfer) paradigms and FZD_5_ as a representative, we recently demonstrated that the FZD/DVL interaction itself is highly dynamic.^20^ WNT addition led to an increase in BRET between luciferase-tagged FZD_5_ and fluorescently labeled DVL/DEP. This signal change was dissected into a recruitment component, i.e., more DVL/DEP molecules interact with FZDs, and a conformational component, i.e., the conformation of the preformed FZD/DVL interface changes. The question remains, however, whether the different FZD paralogs present with the same mode of action regarding their interaction with DVL.

Here, we demonstrate, using the isolated DEP domain of human DVL2, that the majority of FZDs exhibit a high-affinity basal interaction with DVL/DEP. Addition of different WNTs or Norrin led to dynamic changes with distinct kinetic profiles in a unimolecular BRET sensor platform we termed FZD-DEP-Clamps. Besides providing a class-wide measure for FZD/DVL dynamics, the FZD-DEP-Clamps, exemplarily shown for FZD_5_, constitute a unique FZD-focused biosensor suite suitable for high-throughput ligand screening.

Taken together, our data show (i) differences between FZD paralogs in their response to WNT stimulation and (ii) that WNT-induced conformational dynamics occurring at the FZD/DVL interface present a general mechanism, applicable for the majority of FZDs, emphasizing the importance of receptor dynamics for signal initiation in FZDs.

## Experimental Section

### Cloning and Plasmids

Plasmids were generated using standard cloning techniques (restriction cloning and Gibson Assembly). More details can be found in the Supporting Information. The plasmid encoding DEP-Venus was described previously.^20^ A plasmid for the 8X SuperTOPFlash reporter was obtained from Addgene (#12456). A list of all primers used to generate new plasmids can be found in Table S3. All plasmids were verified by Sanger sequencing (Eurofins Genomics).

### Cell Culture and Transfection

HEK293A cells (female origin, Thermo Fisher Scientific, #R70507), ΔFZD_1–10_ HEK293T cells,^22^ and ΔLRP5/6 HEK293T cells^22^ (both kindly provided by Benoit Vanhollebeke, Université de Bruxelles) were cultured in Dulbecco’s modified Eagle’s medium (DMEM, Cytiva, #SH30022.01), which was supplemented with 10% fetal calf serum (FCS; Gibco, #10270106) and 1% penicillin/streptomycin (Gibco, #15070063), in a humidified atmosphere (37 °C, 5% CO_2_).

Transient transfections were performed in suspension using linear polyethylenimine as the transfection reagent (PEI, Alfa Aesar, MW 25,000, stock solution: 1 mg/mL) in a PEI (μL) to DNA (μg) ratio of 5:1. Cells were transfected with 1 μg of total DNA per milliliter of cell suspension. All indicated plasmid amounts refer to the amount used for transfection of 1 mL of cell suspension (density-adjusted). The HEK293A cell line stably expressing the FZD_5_-DEP-Clamp was generated as previously described using 2000 μg/mL G418 sulfate (Gibco, cat.-No.:10131027) as the selection antibiotic.^23^

Cells were routinely checked for mycoplasma contamination using the MycoStrip Mycoplasma Detection Kit (Invivogen, #rep-mys-50) according to the manufacturer’s protocol and were found to be negative.

### Ligands

Recombinant human WNT-3A (#5036-WN-010), human/mouse WNT-5A (#645-WN-010/CF), human WNT-5B (#7347-WN-025), human WNT-16B (#7790-WN-025), and human Norrin (#3014-NR-025) were purchased from R&D Systems/Biotechne. WNTs were resuspended in 0.1% bovine serum albumin (BSA, Sigma-Aldrich #A2153)/Dulbecco’s phosphate buffered saline (DPBS, Hyclone #SH30028), while Norrin was resuspended in sterile 4 mM hydrochloric acid, all at a concentration of 100 μg/mL. Resuspended ligands were stored at 4–8 °C for a maximum of 8 weeks. For some experiments, dilutions of WNT-16B and its vehicle control were subjected to two consecutive heat–freeze cycles (65 and −20 °C for 30 min each) to inactivate the WNT protein. The porcupine inhibitor C59, which was used to suppress endogenous WNT secretion, was obtained from Abcam (#ab142216) and stored at −20 °C as a 5 mM stock solution prepared in DMSO.

### Surface ELISA

HEK293A cells (350,000 cells/mL) were transiently transfected with the indicated plasmids and seeded in a poly-d-lysine (PDL, Sigma-Aldrich, #A3890401)-coated clear 96-well plate (Sarstedt) at a density of 35,000 cells per well. After 40–48 h, the cell culture medium was decanted, 50 μL of a 1:1000-dilution of anti-HA antibody (Abcam, #ab9110) in 1% bovine serum albumin (BSA)/DPBS (+ MgCl_2_ and CaCl_2_, Gibco #14080048) were added and incubated at 4 °C for 1 h. The antibody solution was aspirated and the plate was washed five times with ice-cold 0.5% BSA/DPBS (+ MgCl_2_ and CaCl_2_). Afterward, the plate was incubated with 50 μL of a horseradish-peroxidase conjugated goat anti-rabbit antibody (Thermo Fisher Scientific; #31460, 1:2500 in 1% BSA/DPBS (+ MgCl_2_ and CaCl_2_)) at 4 °C for 1 h. After five washing steps, the washing buffer was decanted and 50 μL of 3,3′,5,5′-tetramethylbenzidine (TMB) liquid substrate (Sigma-Aldrich, #T8665) were added to each well. After 30 min of incubation in the dark, the substrate was acidified with 50 μL of 2 M HCl, followed by measurement of the absorbance at λ = 450 nm in a TECAN Spark microplate reader.

### DEP-mVenus BRET Titration Experiments

HEK293A cells were transfected in suspension (350,000 cells/mL) with a fixed amount of FZD_*x*_-Nluc or mSMO-Nluc (10 ng; 25 ng for FZD_3_, FZD_6_, FZD_6_ Δ559, and human FZD_8_), increasing concentrations of DEP-mVenus and empty pcDNA3.1 vector (add 1 μg DNA per mL cell suspension). The transfected cell suspension (100 μL/well) was transferred to a white, opaque 96-well plate (Greiner Bio-One). After 40–48 h of incubation time (37 °C, 5% CO_2_), cells were washed with HBSS and kept in 90 μL of HBSS until the measurement. First, mVenus fluorescence was measured using a TECAN Spark microplate reader (excitation 485/20 nm, emission 535/25 nm). Then, 10 μL of coelenterazine h solution (Biosynth, final concentration: 5 μM in HBSS) were added to the wells, and after an incubation time of 6 min, BRET emission signals were read (Nluc donor emission between 445 and 485 nm, mVenus acceptor emission between 520 and 560 nm, 100 ms integration time for both channels).

### TOPFlash Assays

To assess β-catenin-dependent signaling, ΔFZD_1–10_ HEK293T cells were transiently transfected in suspension (450,000 cells/mL) with 20 ng of the indicated FZD-DEP-Clamp (wild-type or L445E) and 250 ng of 8X SuperTOPFlash reporter plasmid. The DNA amount was adjusted to 1 μg DNA per mL cell suspension using pcDNA3.1. After 20–24 h, the cells were washed once with HBSS and stimulated overnight with 300 ng/mL WNT-3A or vehicle in starvation medium (serum-free DMEM with 1% penicillin/streptomycin) supplemented with 10 nM of C59. 24 h after stimulation, the transcriptional response was detected with the Dual-Luciferase Assay System (Promega, #E1910) using a slightly modified assay protocol.^20,24^

### FZD-DEP-Clamp Experiments

For transient transfection, HEK293A cells (350,000 cells/mL) were transfected 2 days before the experiment with 20 or 50 ng (for FZD_6_ and FZD_6_ Δ559) of the respective FZD-DEP-Clamp construct (per mL cell suspension). pcDNA3.1 was used to adjust the DNA amount to 1 μg DNA per mL cell suspension. Transfected cells (35,000 cells/well) were seeded in a PDL-coated, white opaque 96-well plate. For *Z*′ factor determination with the stable cell line, HEK293A cells stably expressing the FZD_5_-DEP-Clamp were seeded at a density of 35,000 cells/well in a PDL-coated, white opaque 96-well plate the day before the experiment.

On the day of the experiment, the cells were washed once with HBSS and kept in 90 μL of HBSS (for determination of basal BRET ratio) or 80 μL of 0.1% BSA/HBSS (for ligand stimulation experiments). Next, 10 μL of coelenterazine h (for determination of basal BRET ratio, final concentration: 5 μM in HBSS) or furimazine (for ligand stimulation experiments, NanoBRET Nano-Glo Substrate, Promega, #N1572, prediluted 1:100 in 0.1% BSA/HBSS) were added to the cells and the plate was incubated for 10 min inside the plate reader (prewarmed to 37 °C) before the measurement was started. Three consecutive BRET reads were performed as the baseline read (basal BRET), after which 10 μL of ligand or vehicle (in 0.1% BSA/HBSS) were added and the BRET measurement was continued (for stimulation experiments). For the *Z*′ factor experiments, 48 wells (half plate) were stimulated with either WNT-3A or vehicle. All measurements were performed at 37 °C using a TECAN Spark multimode plate reader (Integration time 100 ms for both emission channels. Nluc bioluminescence detected between 445 and 485 nm, mVenus emission between 520 and 560 nm).

### Data Analysis and Statistics

All experiments were performed at least three times (independent experiments) in technical triplicate. Data measured on the TECAN Spark multimode plate reader were obtained as Microsoft Excel Spreadsheets (Office 365). Data analysis and visualization were performed in GraphPad Prism 9.4.1. More details about the data analysis can be found in the Supporting Information.

Statistical significance between different conditions was assessed using two-tailed Student’s *t* test, one-way analysis of variance (ANOVA), or multiple *t* tests, as detailed in the figure legends. For all statistical tests, *p* < 0.05 was considered significant.

## Results

### Profiling the Constitutive Interaction of FZD Paralogs with the Isolated DEP Domain

**Figure 1:**
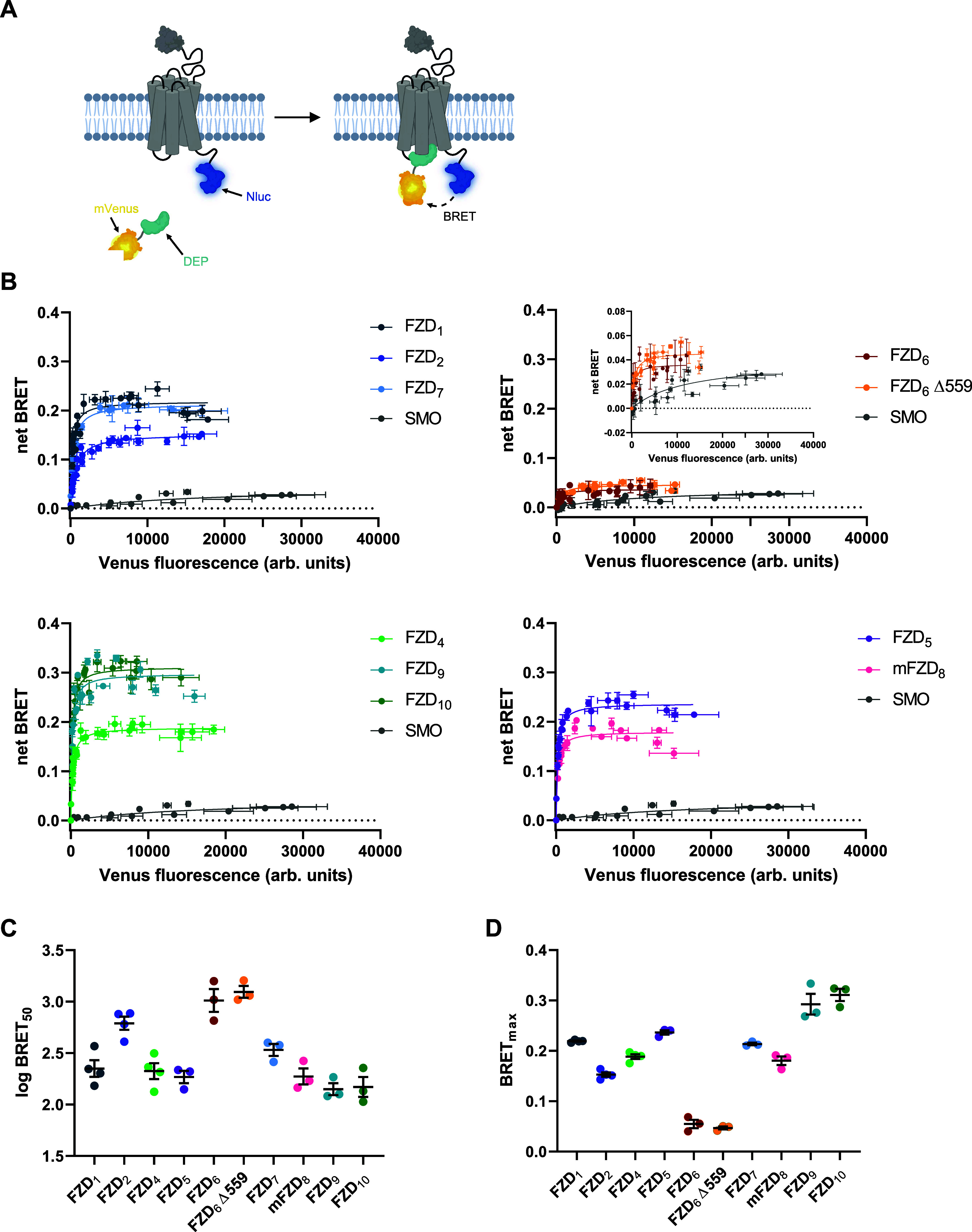
Class-wide profiling of constitutive FZD-DEP interactions. (A) Schematic showing the principle of the direct BRET assay between a C-terminally Nanoluciferase (Nluc)-tagged receptor and the mVenus-tagged isolated DEP domain of human DVL2. Created with biorender.com. (B) Assessment of the constitutive recruitment of DEP-mVenus to FZD_*x*_-Nluc/SMO-Nluc via bioluminescence resonance energy transfer (BRET)-based acceptor titration experiments. Experiments were performed in transiently transfected HEK293A cells. Shown data represent values from three to four independent experiments (superimposed) ± SD, where each experiment was performed in triplicate. (C, D) log BRET_50_ (C) and BRET_max_ (D) values extracted from DEP titration experiments shown in (B). Data represent mean values ± SEM from three to four experiments performed in triplicate.

Initially, we investigated the basal, ligand-independent interaction between the 10 FZD paralogs and the isolated DEP domain of DVL2 adapting a direct BRET-based approach.^20^ We focused on DEP, as it represents the key domain for FZD recognition.^17,25^ The assay setup is based on a C-terminally Nanoluciferase (Nluc)-tagged receptor and the mVenus-tagged DEP domain of DVL2 (Figure 1A).^20^ To quantify the basal recruitment of DEP to FZDs, we performed BRET acceptor titration experiments by gradually increasing the amount of the BRET acceptor plasmid (DEP-mVenus), while keeping the BRET donor plasmid amount (FZD_*x*_-Nluc) constant. Membrane expression of all receptor-Nluc constructs was confirmed by surface enzyme-linked immunosorbent assay (ELISA, Figure S1A and Table S1). In BRET titration experiments, we obtained hyperbolic curves for most FZD paralogs indicative of a high-affinity and specific constitutive interaction (see Figure 1B). As expected, experiments with SMO resulted in a straight line (nonspecific interaction), as the observable BRET increase is only mediated by random collision of donor molecules with the increasing amount of acceptor molecules. Notably, FZD_3_ showed a very low-affinity or close to nonspecific interaction with DEP in our assay (see Figure S1B). Moreover, while mouse FZD_8_ (mFZD_8_) exhibited a high affinity toward DEP (see Figure 1B), the human ortholog (human FZD_8_) did not interact specifically (see Figure S1B). As both FZD_3_ and human FZD_8_ were reported to induce DVL-dependent processes, such as planar cell polarity-like signaling or WNT/β-catenin signaling, respectively,^26−28^ we cannot exclude, however, that these interactions are either not captured in our assay due to methodological limitations or are mediated by other domains of DVL. All other FZDs bound DEP with high affinity displaying log BRET_50_ values in the same range, with FZD_2_ and FZD_6_ exhibiting slightly lower affinities (i.e., higher log BRET_50_ values, see Figure 1C). Similarly, BRET_max_ values (maximum BRET shift) were in a similar range, with FZD_6_ being the only paralog displaying a substantially lower BRET_max_ value (see Figure 1D). It should be noted that—to a large degree—log BRET_50_ and BRET_max_ values from DEP titration experiments are independent of receptor expression levels suggesting they are indeed receptor-specific parameters.^24^ As the C-terminus of FZD_6_ is much longer than that in other FZDs (except FZD_3_), we reasoned that this could explain the strongly reduced BRET_max_ value. Theoretically, a lower BRET_max_ value can be explained by a less favorable orientation of the donor toward the acceptor and/or a larger distance between the entities fused to the BRET pair (FZD_6_ and DEP). Therefore, we generated a C-terminally truncated version, FZD_6_ Δ559, with the C-terminus containing the same number of residues as the C-terminus of FZD_5_. However, FZD_6_ Δ559 essentially retained the DEP-binding properties (log BRET_50_ and BRET_max_) of full-length FZD_6_ (see Figure 1), implying that the obtained differences are indeed receptor-specific.

### Validation of Unimolecular Sensors to Investigate the Dynamics of FZD-DEP Interactions

**Figure 2:**
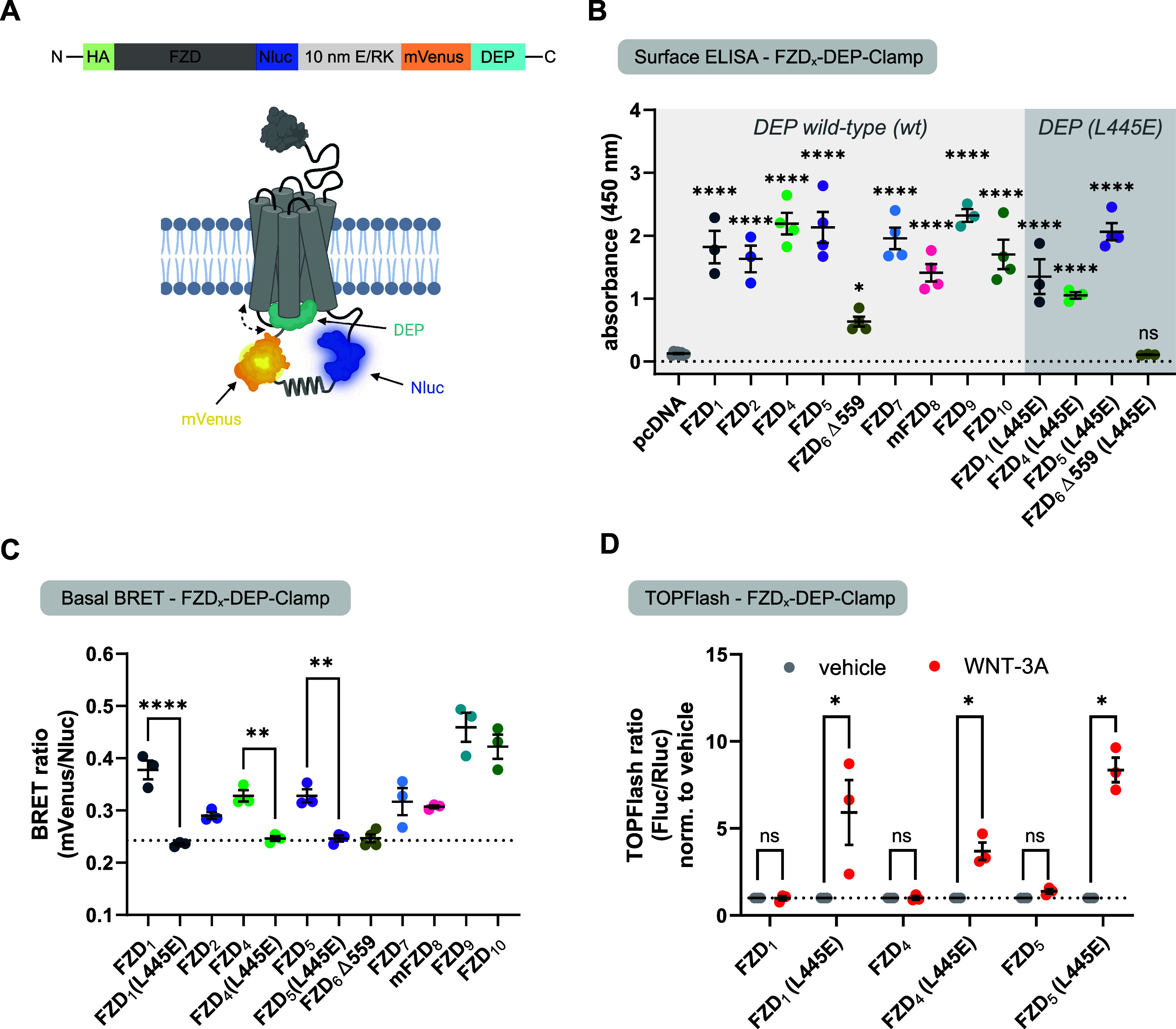
Basal validation of unimolecular FZD-DEP biosensors. (A) Architecture of the unimolecular FZD-DEP sensors, consisting of the respective FZD sequence, Nanoluciferase (Nluc), a 10 nm E/RK linker, mVenus, and the DEP domain of human DVL2. Created with biorender.com (B) Surface expression analysis of unimolecular FZD-DEP biosensors as determined by surface ELISA in transiently transfected HEK293A cells. Data show mean values ± SEM from three to four (eight for pcDNA) independent experiments, performed in triplicate. Statistical significance compared to an empty-vector transfection (pcDNA) was assessed using one-way ANOVA followed by Fisher’s Least Significant Differences analysis. (C) Basal BRET ratios of unimolecular FZD-DEP biosensors (wild-type (wt) or L445E) in the absence of ligand. The black dashed line represents the average BRET ratio of the three surface-expressed FZD-DEP (L445E) sensors. Experiments were performed in transiently transfected HEK293A cells. Data show mean values ± SEM from three to four independent experiments performed in triplicate. Statistical significance was assessed using one-way ANOVA followed by Sidák’s multiple comparison test. (D) TOPFlash response of selected unimolecular FZD-DEP sensors upon stimulation with vehicle or WNT-3A (300 ng/mL for FZD_1_ and FZD_5_; 1 μg/mL for FZD_4_). Experiments were conducted in transiently transfected ΔFZD_1–10_ HEK293T cells. Data show mean values (normalized to vehicle) ± SEM from three independent experiments (each performed in triplicate). Statistical significance between vehicle- and WNT-3A treated conditions was assessed using multiple *t* tests (two-tailed). ns: not significant; *: *p* < 0.05; **: *p* < 0.01; ****: *p* < 0.0001.

The WNT-induced BRET increase in the direct BRET setup was shown to be a composite of DEP recruitment to FZD and a conformational rearrangement in the preformed FZD/DEP complex.^20^ To separate the conformational component, we recently developed a unimolecular biosensor for FZD_5_, which we termed FZD_5_-DEP-Clamp.^20^ Originally inspired by receptor-G protein sensors called SPASMs,^29^ this sensor directly connected FZD_5_-Nluc with DEP-mVenus via a 10 nm α-helical linker (E/RK linker) (see Figure 2A). Due to the high basal affinity of the DEP domain to the FZD_5_ core, this sensor is in a closed state, i.e., DEP is bound to FZD_5_, in the absence of WNT. This tight closure is the prerequisite that enabled us to confidently assess WNT-induced dynamics in the FZD/DVL interface justifying to name the sensor “FZD-DEP-Clamp”.^20^ Here, we adapted this sensor principle for a family-wide assessment of ligand-induced dynamics. We cloned analogous unimolecular FZD-DEP sensors for all FZDs showing specific interactions with DEP-mVenus in our direct BRET setup (Figure 1).

For FZD_6_, we focused on C-terminally shortened FZD_6_ (FZD_6_ Δ559) to exclude the potential impact of an excessively long C-terminus. After confirming that all FZD-DEP wild-type (wt) sensors were localized to the plasma membrane (Figure 2B, left, and Table S2), we validated the sensors by determining the BRET ratios in the absence of ligand stimulation (Figure 2C). The observed basal BRET values correlated very well with the BRET_max_ values from the respective FZD_*x*_-Nluc DEP-mVenus titrations (see Figure S2), suggesting that linking FZD and DEP in the unimolecular sensor does not alter the paralog-specific transducer coupling. To verify that the unimolecular FZD-DEP sensors are “clamped”, as in the case of FZD_5,_^20^ we generated sensor variants for a representative member of each FZD homology cluster (FZD_1_, FZD_4_, FZD_6_ Δ559) carrying a mutation in the finger loop of the DEP domain equivalent to the L445E mutation of DVL2. This mutation abolished the interaction between FZD and DEP in different experimental paradigms.^18,20,25^ Introducing the L445E mutation into the DEP domain of the biosensor should force it into an open state; i.e., DEP should not be able to engage the FZD anymore, resulting in a decreased basal BRET ratio. While the L445E variants of FZD_1_, FZD_4_, and FZD_5_^20^ were localized at the plasma membrane, the FZD_6_ Δ559-DEP (L445E) sensor did not surpass the detection threshold of the surface ELISA (see Figure 2B, right, and Table S2) and was therefore excluded in subsequent experiments. Expectedly, the basal BRET ratio of the FZD_1/4/5_ sensors harboring the L445E mutation was notably reduced to similar, significantly lower levels compared to their respective wild-type counterparts (Figure 2C). This validates that the mutated DEP domain is not able to interact with the receptor core, while, on the contrary, the wild-type DEP domain in the unimolecular sensors is tightly bound or “clamped”.

This conclusion was further strengthened by assessing the WNT-3A-induced and β-catenin-mediated transcriptional activities in a luciferase-based reporter gene assay (TOPFlash). For robust β-catenin-dependent signaling, it is necessary that (endogenous or overexpressed) DVL can functionally interact with the transmembrane core of FZDs.^25,30^ In the case of the FZD-DEP (wt) sensors, we were not able to detect a robust TOPFlash signal upon sensor overexpression and WNT-3A stimulation, as the sensor’s DEP domain, which on its own is not capable of transducing the signal, clings to the receptor occupying the transducer interaction site (see also high basal BRET in Figure 2C). Thus, access of endogenous DVL molecules to the receptor is sterically hindered preventing a WNT-induced TOPFlash signal. In contrast, a substantial WNT-induced TOPFlash response could be observed for the sensor analogs harboring the L445E mutation in their DEP domain (Figure 2D) suggesting that endogenous DVL was still able to access the receptor core. This observation proves, in contrast to the FZD-DEP (wt) sensors, that the mutated sensor’s DEP domain does not occupy the transducer binding site of the sensor FZD.

Extrapolating our results to all paralogs, we claim that all unimolecular FZD-DEP (wt) sensors are FZD-DEP-Clamps. We surmise that for all FZD-DEP-Clamps, potential changes in BRET upon ligand stimulation reflect a conformational change within the FZD-DEP interface of these biosensors instead of agonist-induced recruitment of the DEP domain to FZD. For FZD_6_ Δ559, we could not define whether the sensor is in a closed or an open state due to the very low basal BRET ratio and the lack of surface expression of its FZD-DEP (L445E) variant.

### Ligand Profiling of FZD-DEP-Clamp Sensors

**Figure 3:**
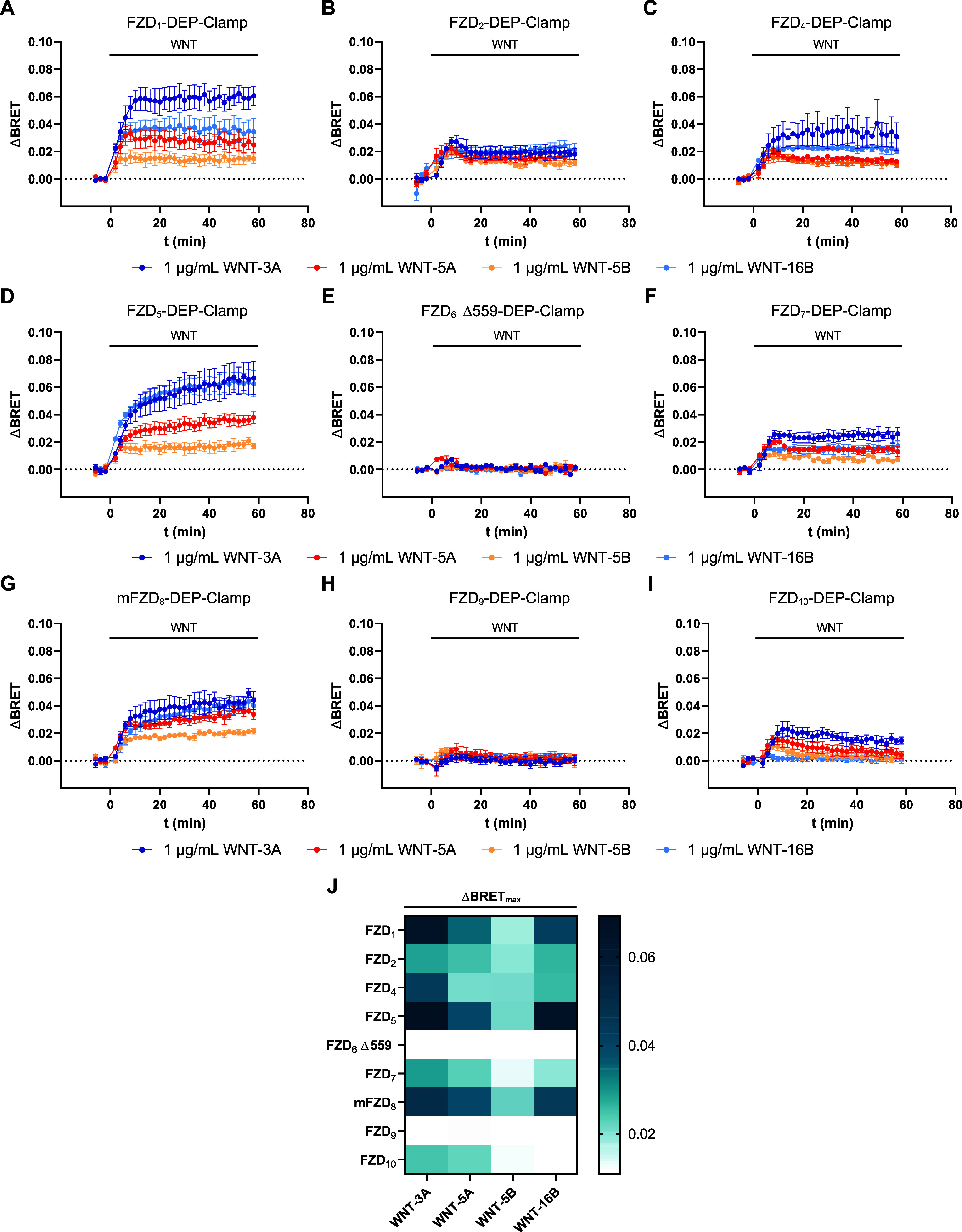
Kinetic profiles of unimolecular FZD-DEP (wt)-Clamps upon WNT stimulation. (A–I) Kinetic BRET responses of the FZD_1_- (A), FZD_2_- (B), FZD_4_- (C), FZD_5_- (D), FZD_6_ Δ559- (E), FZD_7_- (F), mFZD_8_- (G), FZD_9_- (H), or FZD_10_-DEP-Clamp (I) upon stimulation with WNT-3A, WNT-5A, WNT-5B, or WNT-16B. Experiments were conducted in HEK293A cells transiently transfected with the indicated FZD-DEP-Clamp. Data present mean values ± SEM from three to five independent experiments performed in triplicate. (J) Heatmap depicting the peak ΔBRET values (ΔBRET_max_) for every FZD-DEP-Clamp–WNT combination.

We reasoned that our newly generated FZD-DEP-Clamp sensor toolbox is well suited to investigate WNT-FZD selectivity and FZD-paralog- and ligand-dependent differences in dynamic responses. To this end, we recorded kinetic traces of all FZD-DEP-Clamp sensors in response to stimulation with four different WNTs: WNT-3A, WNT-5A, WNT-5B, and WNT-16B (Figure 3A–I). While WNT-3A is a well-described activator of β-catenin signaling, WNT-5A, WNT-5B, and WNT-16B are mostly involved in β-catenin-independent signaling.^31−35^

All FZD-DEP-Clamps, except for the FZD_6_ Δ559- and the FZD_9_-DEP-Clamp (see also Figure S3), responded to several of the tested WNTs with a dynamic increase in BRET (see Figure 3J). A notable exception was the FZD_10_-DEP-Clamp, which did not respond to WNT-16B. The observed signals were WNT-specific, as experiments with WNT-16B dilutions subjected to heat inactivation, (i.e., destruction of pharmacologically active WNT), failed to induce BRET responses at various FZD-DEP-Clamps (see Figure S4A). Moreover, WNT stimulation of an FZD_5_-DEP-Clamp lacking the extracellular CRD (i.e., the WNT binding site) did not lead to BRET changes compared to the vehicle control, further proving the specificity and validity of the observed signal. It should be noted that the removal of the CRD did not affect the basal interaction of FZD_5_ with DEP (see Figure S4B–F).

It became evident that different FZD-DEP-Clamps showed distinct kinetic behaviors. For several paralogs (FZD_1_/FZD_5_/FZD_7_/mFZD_8_) (Figure 3A), WNT stimulation resulted in a BRET increase reaching a stable plateau, while for the FZD_2_- and the FZD_10_-DEP-Clamp (Figure 3B), the ΔBRET value slowly decreased after reaching a peak. Moreover, we observed differences in maximal ΔBRET changes between the different WNTs for some FZD-DEP-Clamps. These, however, are difficult to interpret with respect to biological implications, as the various recombinant WNT preparations show varying activity and purity, which contributes to the observed differences.

Co-receptors such as LRP5/6 play an important role in the specification and initiation of WNT signaling.^10,11,36^ At the example of WNT-3A and LRP5/6, we wanted to investigate whether the WNT-induced responses we observed for the FZD-DEP-Clamps are impacted by the presence of co-receptors. To this end, we transfected selected FZD-DEP-Clamps into HEK293 LRP5/6-knockout cells (ΔLRP5/6 HEK293) and stimulated them with WNT-3A. We could not detect any differences in the kinetic traces between regular HEK293 cells and ΔLRP5/6 HEK293 cells (Figure S5A), suggesting that WNT-induced conformational dynamics at the FZD-DEP interface are independent of LRP5/6.

Norrin is an atypical FZD ligand indispensable for the development of retinal vasculature and maintenance of the blood-brain barrier.^37,38^ It is a homodimeric protein comprising a cystine-knot motif and being structurally unrelated to WNTs.^39,40^ In contrast to WNTs, Norrin displays selectivity for the CRD of FZD_4_ without cross-reactivity to any other FZD.^37,41^ Notably, Norrin led to a very robust increase in BRET when added to the FZD_4_-DEP-Clamp, whereas the FZD_5_-DEP-Clamp did not respond, confirming the selectivity profile. Performing Norrin experiments in ΔLRP5/6 HEK293 cells resulted in a similar kinetic trace for the FZD_4_-DEP-Clamp showing a consistently higher plateau value, which contrasted with our previous observation for WNTs (Figure S5B).

### Assessment of High-Throughput Compatibility of the FZD_5_-DEP-Clamp

In contrast to other GPCRs, the pharmacology of FZDs is still scarce, despite the apparent attraction of FZDs as a potential drug target. While some efforts and improvements have been made,^42−47^ progress is hampered by the lack of assays robust enough to fulfill the requirements for high-throughput screening campaigns.

**Figure 4:**
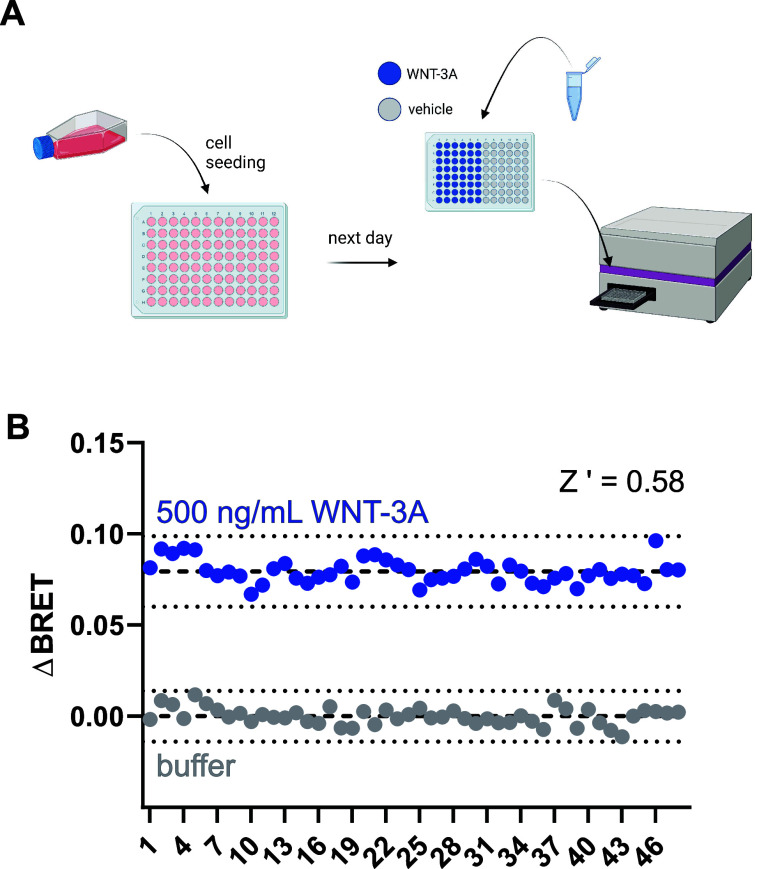
Determination of the *Z*′ factor for the stably expressed FZD_5_-DEP-Clamp. (A) Schematic showing the experimental setup of *Z*′ factor determination. Created with biorender.com. (B) Representative results from one plate treated with 500 ng/mL WNT-3A or vehicle. Experiments were performed in HEK293A cells stably expressing the FZD_5_-DEP-Clamp.

Due to the robust signal obtained in kinetic measurements, we reasoned that the FZD-DEP-Clamp is sensitive enough for high-throughput applications. Additionally, a unimolecular sensor encoding all elements on a single plasmid improves the robustness of the assay compared to a multiplasmid transfection.^48^ As a proof of concept, we generated a HEK293A cell line stably expressing the FZD_5_-DEP-Clamp and tested its high-throughput compatibility by calculating the *Z*′ factor.^49^ This measure is typically used to assess whether a test system is sensitive and robust enough (*Z*′ has to be larger than 0.5) to be used for high-throughput screening campaigns. To this end, the stable cell line was stimulated with 500 ng/mL WNT-3A or vehicle control (Figures 4 and S6). We determined a *Z*′ factor of 0.54 ± 0.04 (mean ± SD) for our stable cell line, thus surpassing the assay requirements (*Z*′ > 0.5) for screening campaigns.

## Discussion

In this work, we present a class-wide characterization of the FZD-DVL interaction from two different angles, for both of which we use the isolated DEP domain as a proxy for full-length DVL. We use a bimolecular NanoBRET setup to establish the ligand-independent interaction profile between all FZDs and DEP and create a unimolecular sensor toolbox, the FZD-DEP-Clamps, to probe the ligand-induced conformational dynamics at the FZD/DEP interface. The FZD_5_-DEP-Clamp surpassed the assay sensitivity requirements for high-throughput campaigns (*Z*′ > 0.5) presenting a unique, FZD-focused readout sensitive enough for screening of larger ligand libraries facilitating the discovery of FZD-targeting molecules in the future.

Regarding the ligand-independent interaction profile, we observed that all FZDs but FZD_3_ and human FZD_8_ interacted with DEP in our setup with very high affinity further supporting the notion that FZDs couple to DVL as their main transducer.^24^ Among the interacting FZDs, FZD_6_ was a clear outlier as it showed lower affinity and strongly reduced BRET_max_ values compared to all other paralogs. Since FZD_6_ also differs in terms of its signaling profile, as it is typically incapable of inducing WNT/β-catenin signaling,^7,50^ it is tempting to speculate that its divergent signaling preference arises from a distinct mode of interaction with DVL.

The sensor toolbox of FZD-DEP-Clamps for FZD_1, 2, 4, 5, 7, 8, 9, 10_ allowed us to directly compare ligand-induced effects on the conformation of the FZD/DEP interface. Most FZD-DEP-Clamps responded promiscuously to all four tested WNTs (WNT-3A, WNT-5A, WNT-5B, and WNT-16B) with an increase in BRET (Figure 3). Notably, WNT-16B elicited robust responses with several FZD-DEP-Clamps even though no core dynamics could be detected with cpGFP-based conformational FZD sensors.^51^ Furthermore, the observed BRET responses showed paralog-distinct kinetics, in terms of both their speed and their curve shape. These might, for example, originate from differences in receptor turnover in response to agonist stimulation. While it is difficult and speculative to put this into a biological context based on our current data, it still underlines intrinsic differences among the 10 FZD paralogs.

Neither FZD_6_- (or FZD_6_ Δ559) nor FZD_9_-DEP-Clamp showed any change in BRET in response to any of the tested WNTs. While this confirms the special role and behavior of FZD_6_, this finding was particularly surprising for FZD_9_, as it was the FZD-DEP-Clamp with the highest basal BRET value. One potential explanation for the lack of dynamic BRET response might be the actual lack of agonist binding, as there are discrepancies in the literature on whether FZD_6_ and FZD_9_ can bind certain WNTs or not.^28,51^ Another possible explanation for the lack of signaling would be the functional selectivity of the WNTs, as FZD_6_ and FZD_9_ were both shown to couple to or signal via heterotrimeric G proteins upon WNT stimulation.^42,52−54^ While we cannot rule out any of the aforementioned explanations, we propose that the lack of signal rather reflects receptor-dependent behavior given that a wide array of WNT/FZD combinations showed a robust dynamic response independent of whether they feed into β-catenin-dependent or -independent signaling.

Mechanisms of WNT/FZD signal initiation and specification remain an unresolved enigma in the field. In this context, the FZD-DEP-Clamps offer a promising perspective to integrate two seemingly opposing paradigms in WNT signaling: FZD-intrinsic receptor dynamics and signalosome formation. The latter refers to the formation of a higher-order complex or signalosome consisting of FZD, WNT, and LRP5/6, resulting in increased local concentration of DVL at the membrane.^55,56^ Signalosome formation is thought to be both necessary and sufficient for the initiation of WNT-induced β-catenin-dependent signaling while being independent of intrinsic receptor dynamics. As the unimolecular biosensors capture agonist-induced conformational dynamics in the FZD-DEP interface, we demonstrate here that the majority of FZDs are in fact capable of propagating a conformational change across the membrane when subjected to various ligands. Importantly, the conformational rearrangements of the FZD-DEP-Clamps upon WNT stimulation also occurred independently of the presence of LRP5/6, as shown by experiments in ΔLRP5/6 HEK293 cells. This is in agreement with previous reports showing that neither CRD nor receptor core dynamics were affected upon blocking the WNT-3A binding site on LRP5/6 by dickkopf-1 (DKK1).^51,57^

Norrin, a structurally distinct FZD_4_-selective ligand typically activating β-catenin signaling, induced a robust BRET change in the FZD_4_-DEP-Clamp that was reminiscent of the WNT-induced response. It was previously reported that Norrin addition led to an increase in BRET between a luciferase-tagged FZD_4_ and fluorescently labeled DVL2, which was interpreted as additional recruitment of DVL to the plasma membrane.^58^ As the FZD-DEP-Clamps report on intrinsic rearrangements, we expand on this hypothesis by adding that Norrin elicits conformational changes in FZDs particularly in the FZD/DEP or FZD/DVL interface. This is in agreement with previous HDX/MS measurements showing that the intracellular loop 3 of FZD_4_ is dynamic in response to Norrin.^58^ Notably, the Norrin-induced responses at the FZD_4_-DEP-Clamp were maintained in the absence of LRP5/6. In contrast to WNTs, Norrin acts as a homodimer stabilizing a receptor complex with different stoichiometry.^40,59^ While we can only speculate whether the distinct architecture of the Norrin-FZD-LRP complex is responsible for the increase in the Norrin-induced BRET_max_ in the absence of LRP5/6, these observations nevertheless indicate that LRP5/6 is not required for Norrin-induced changes in the FZD/DEP complex.

The concept of agonist-induced conformational changes in the receptor-transducer interface is clearly reminiscent of the classical ternary complex model for GPCRs, where agonist and transducer interact in a bidirectional, transmembrane allosteric interaction resulting in a fully active and G protein-coupled GPCR.^60,61^ The observed BRET dynamics in the FZD-DEP-Clamp sensors in response to WNT or Norrin strongly argue for an allosteric cooperativity between agonist binding and DEP coupling. In this context, it is important to mention that affinity changes in a reconstituted FZD-DEP system could neither be captured with WNT (FZD_4, 5_) nor with Norrin (FZD_4_) addition.^62^ These findings with purified proteins and a reconstituted system were interpreted as proof for the absence of transmembrane allostery, which stands in stark contrast to our data in living cells.^63^

## Conclusions

In summary, we mapped the constitutive and ligand-induced interaction between the DEP domain of DVL2 and the 10 FZDs in a receptor family-wide manner. In the absence of any ligands, most FZD paralogs showed very similar behavior displaying a high propensity to bind the DEP domain. While most tested WNT/FZD combinations led to conformational dynamics in unimolecular sensors, distinct profiles could be observed for the different FZD paralogs with respect to their kinetics and their selectivity profile (or the lack of the latter). These clear-cut differences emphasize that the 10 FZD paralogs vary in terms of the underlying mechanisms of FZD-DVL communication. Future studies will specify differences between the members of the FZD family and the understanding of their peculiarities. Importantly, the FZD-DEP-Clamp sensor toolbox provides unprecedented possibilities for future drug discovery efforts presenting a high-throughput-compatible and FZD-focused sensor platform.

## Supporting Information Available

Details about cloning procedures and data analysis; surface expression analysis of receptor-Nluc constructs, and DEP titration experiments with FZD_3_-Nluc/hFZD_8_-Nluc (Figure S1); correlation plot between BRET_max_ values and basal BRET (Figure S2); validation and WNT stimulation of full-length (FL) FZD_6_-DEP-Clamp (Figure S3); proof of specificity for WNT-induced BRET responses seen for the FZD-DEP-Clamp sensors (Figure S4); impact of LRP5/6 on the WNT- or Norrin-induced BRET responses at selected FZD-DEP-Clamps (Figure S5); *Z*′ factor determination and time-dependence of the *Z*′ factor for the FZD_5_-DEP-Clamp (Figure S6); and *p*-values from surface ELISA experiments and primers used in the study (Tables S1–S3) (PDF)

## Abbreviations

BRET: bioluminescence resonance energy transfer
DEP: Dishevelled, Egl-10, Pleckstrin domain
DVL: Dishevelled
FZD: Frizzled
GPCR: G protein-coupled receptor
HDX/MS: hydrogen–deuterium exchange mass spectrometry
LRP5/6: low-density lipoprotein receptor-related proteins 5/6
SMO: Smoothened
WNT: Wingless/Int-1
