# Supplementary material for: Class-Wide Analysis of Frizzled-Dishevelled Interactions Using BRET Biosensors Reveals Functional Differences among Receptor Paralogs
Source: ACS Sens. 2024 Aug 30;9(9):4626–36. doi: 10.1021/acssensors.4c00806 (PMC11443525; doi:10.1021/acssensors.4c00806)
Supplement: Supplementary file 1 — se4c00806_si_001.pdf [file se4c00806_si_001.pdf]

Supporting Information

Class-wide analysis of Frizzled-Dishevelled interactions using BRET biosensors reveals functional differences among receptor paralogs

Authors: Lukas Grätz[a], Jan Hendrik Voss[a], Gunnar Schulte[a,*]

Affiliations:

[a]Karolinska Institutet, Dept. Physiology & Pharmacology, Sec. Receptor Biology & Signaling, Biomedicum, S-17165 Stockholm, Sweden

*To whom correspondence should be addressed: gunnar.schulte@ki.se

Supplementary Methods

Cloning details

Plasmids encoding HA-FZD_4-Nluc, HA-FZD_5-Nluc and HA-FZD_{10}-Nluc (all with a 10 amino acid linker -GSSLDGGGGS- between the receptor and Nluc) were described previously[1]. Plasmids for the other FZD_x-Nluc as well as mSMO-Nluc constructs (note that the constructs do not contain the native signal peptides) were generated by replacing the FZD-encoding nucleotide sequence in HA-FZD_4-Nluc via *BamH*I and *Xba*I restriction sites (for FZD_1, FZD_7 and human FZD_8) or Gibson Assembly (FZD_2, FZD_3, ΔCRD-FZD_5, FZD_6, FZD_6 Δ559, mouse FZD_8 and FZD_9). The following templates were used for amplification of the receptor-encoding sequences: FZD_1, FZD_3, FZD_6, FZD_7, human FZD_8 were amplified from HiBiT-FZD_x[2], the sequence for mouse SMO from mouse SMO-Rluc8 (kindly provided by Nevin A. Lambert, Augusta University). Analogously, the sequence for a FZD_5 lacking the extracellular CRD (ΔCRD-FZD_5 [aa G180-V585]) and a C-terminally truncated FZD_6 (FZD_6 Δ559 [aa H19-G559]) were amplified from HiBiT-FZD_5 and HiBiT-FZD_6, respectively. pCMV6-XL4 FZD_2 (Origene, #SC127603) served as a template for the nucleotide sequence of FZD_2, while pCS2+ mouse FZD_8 (kindly provided by Stephane Angers, University of Toronto) was used as a template for the mouse FZD_8 sequence. For FZD_9, four point mutations originally present in the commercially available plasmid, were curated in the HiBiT-FZD_9 construct[2], which subsequently served as a template for amplification of the FZD_9-encoding sequence.

The unimolecular FZD-DEP-Clamp sensors were cloned by exchanging the receptor sequence in the plasmid encoding the FZD_5-DEP-Clamp[3] with the respective FZD sequence (HA-FZD-Nluc constructs as templates) via Gibson Assembly. The plasmids for the FZD_1-, FZD_4 and FZD_6 Δ559-DEP (L445E) sensors, which carry a mutation in the finger loop of the DEP domain abolishing the interaction of DEP with FZD, were cloned analogously by exchanging the receptor sequence in the FZD_5-DEP (L445E) sensor via Gibson Assembly[3]. To generate FZD_5-DEP-Clamp (TK), the sequence for the human thymidine kinase promoter (TK) was amplified from pRL-TK (Promega) and used to replace the CMV promoter present in the original FZD_5-DEP-Clamp plasmid via Gibson assembly.

Data analysis

Raw BRET ratios were defined as the ratio of acceptor fluorescence (mVenus) over donor bioluminescence (Nluc).

For each BRET acceptor titration experiment, mVenus fluorescence (before substrate addition) and BRET (after substrate addition) measurements were performed three times per plate and averaged. The averaged fluorescence and BRET values were then corrected for the background fluorescence/BRET (0% DEP-mVenus control included in each titration, baseline-corrected BRET = net BRET). Net BRET datasets were analyzed using non-linear regression (one site model – specific binding) or linear regression models implemented in GraphPad Prism 9. The used model was selected for each independent experiment based on extra-sum-of-squares F tests ($p < 0.05$).

For ligand stimulation experiments with the FZD-DEP-Clamp sensors, the average of the first three reads (baseline read before agonist stimulation, basal BRET) was subtracted for every well (baseline-corrected BRET) to correct for variations in the baselines between different wells. Next, for every timepoint, the average of the baseline-corrected BRET ratios from vehicle-stimulated wells was subtracted from the baseline-corrected BRET ratios from ligand-stimulated wells to determine the actual net effect of the ligand (ΔBRET values). The maximal ΔBRET value was determined for each independent experiment using GraphPad Prism. Values from independent experiments were then averaged and used to generate the heatmap in **Figure 3J**.

Z'-factors were calculated according to a previous publication[4] based on the following equation

$$Z' = 1 - \frac{3 \times (\sigma[WNT] + \sigma[vehicle])}{|\mu[WNT] - \mu[vehicle]|}$$

where σ and μ are the standard deviations and average ΔBRET values induced by 500 ng/mL WNT-3A or vehicle control, respectively.

TOPFlash ratios were defined as the ratio of bioluminescence originating from Fluc (transcriptional activation) over bioluminescence originating from Nluc (from FZD-DEP-Clamps, proxy for transfection efficiency) and calculated for every well. The obtained ratios were then corrected by normalizing to the average of the wells stimulated with vehicle control for every transfection condition.

Figure S1

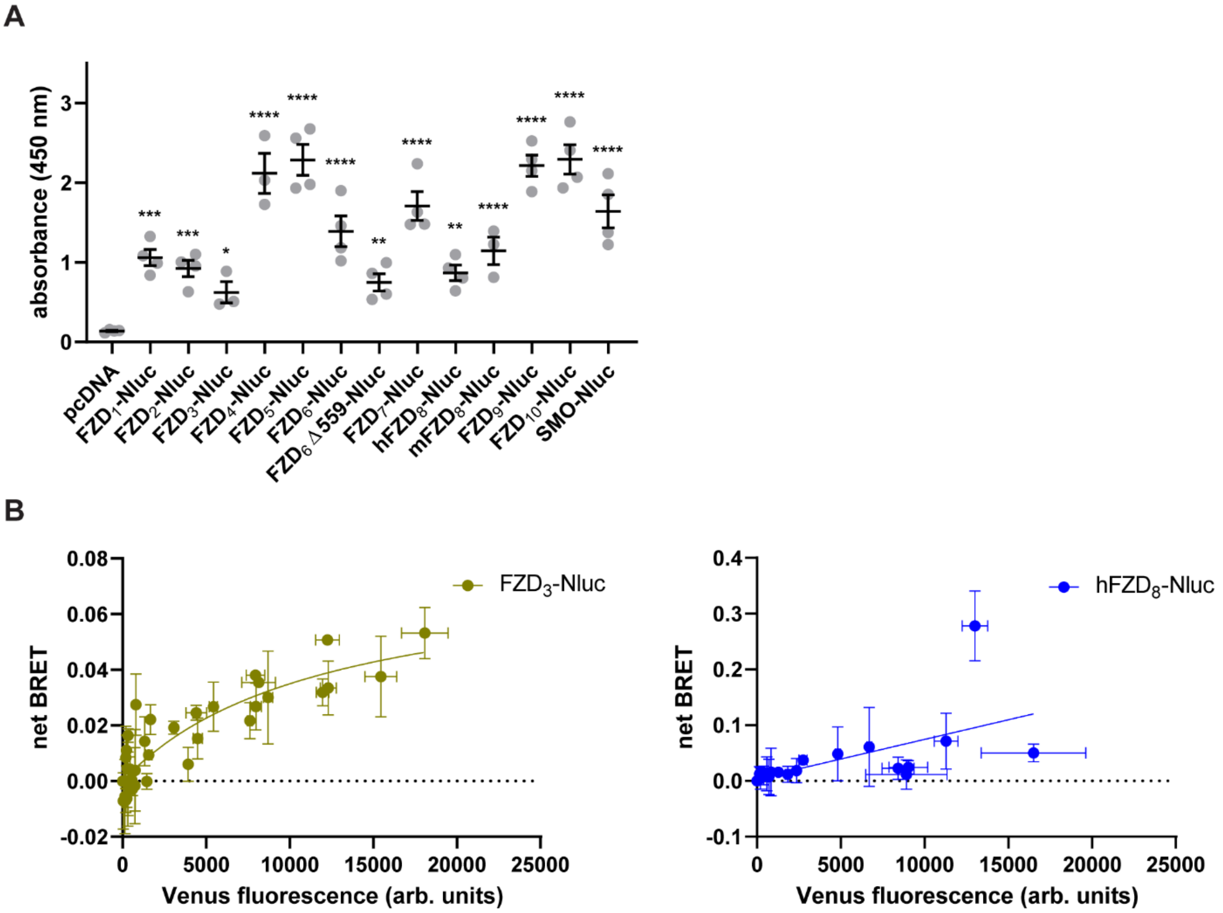

Figure S1 (related to Figure 1): Surface expression analysis of receptor-Nluc constructs and DEP titration experiments with FZD$_3$-Nluc/hFZD$_8$-Nluc.

(**A**) Surface expression analysis of receptor-Nluc constructs. Surface expression was assessed via surface ELISA using an antibody directed against an N-terminally located HA tag. Experiments were performed in HEK293A cells transiently transfected with the indicated receptor-Nluc constructs. Data shown represent mean values ± SEM from three to four independent experiments performed in triplicate. Statistical significance compared to a control transfection with empty pcDNA3.1 was assessed using one-way ANOVA followed by Fisher's LSD test. *: $p < 0.05$; **: $p < 0.01$; ***: $p < 0.001$; ****: $p < 0.0001$. Corresponding p-values can be found in Supplementary Table S1.

(**B**) BRET-based DEP titration experiments with FZD$_3$-Nluc and human FZD$_8$-Nluc (hFZD$_8$-Nluc). Experiments were performed in HEK293A cells transiently transfected with a constant amount of FZD$_3$-Nluc or hFZD$_8$-Nluc and increasing amounts of DEP-mVenus. Shown data are superimposed data points from three (hFZD$_8$) or four (FZD$_3$) independent experiments performed in triplicate. Error bars in x- and y-direction represent SD.

Figure S2

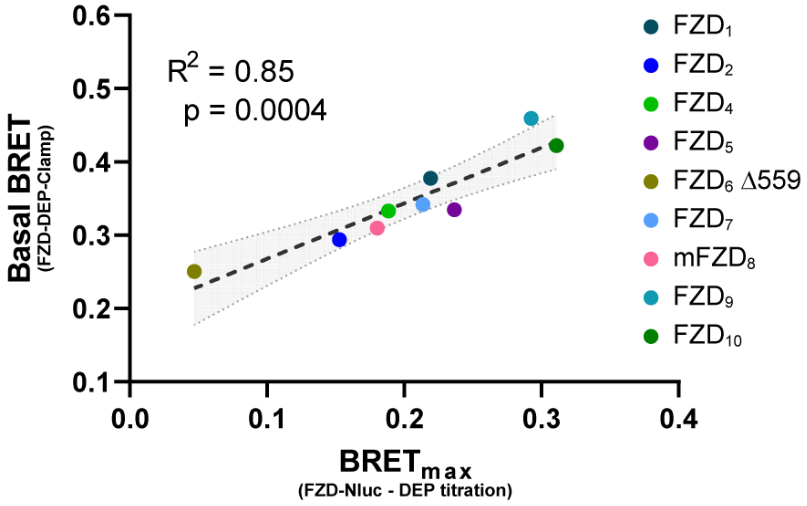

Figure S2 (related to Figure 2): Correlation plot between $BRET_{max}$ values and basal BRET.

Shown is a scatter plot displaying the correlation between the $BRET_{max}$ values extracted from DEP titration experiments (performed with FZD_x-Nluc and DEP-Venus, see Figure **1B**) and the basal BRET values of the FZD_x-DEP-Clamp sensors (see **Figure 2C**). Data were analyzed by linear regression and shown are the linear fit (black dashed line) with the 95% confidence interval.

Figure S3

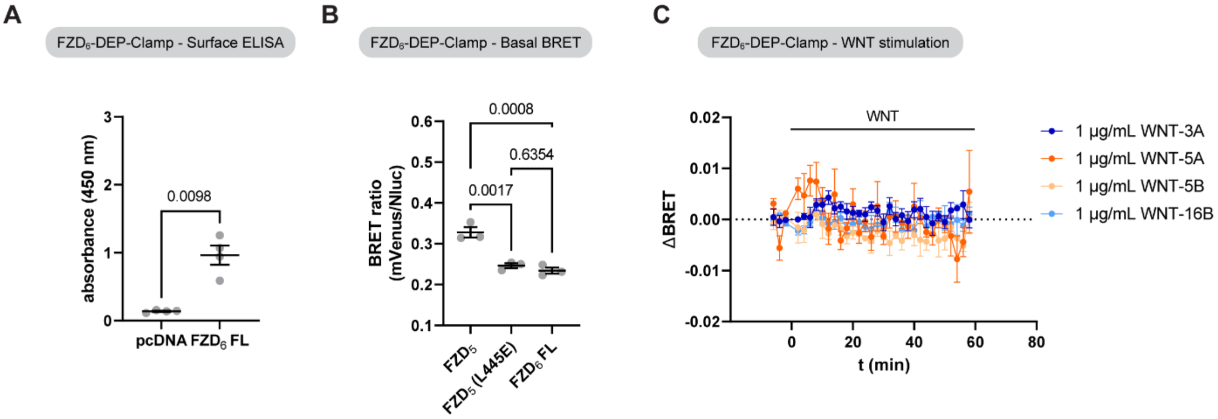

Figure S3 (related to Figure 3): Validation and WNT stimulation of full-length (FL) FZD6-DEP-Clamp.

(**A**) Surface expression analysis of full-length (FL) FZD6-DEP-Clamp. Plasma membrane expression was assessed via surface ELISA using an antibody directed against the N-terminally located HA tag. Experiments were performed in HEK293A cells transiently transfected with the indicated plasmid. Data represent mean values ± SEM from four experiments performed in triplicate.

(**B**) BRET ratio of full-length (FL) FZD6-DEP-Clamp in the absence of ligand. Experiments were performed in HEK293A cells transiently transfected with the indicated constructs. Data show mean values ± SEM from three independent experiments performed in triplicate. Data for FZD5-DEP-Clamp ("closed form") and FZD5-DEP (L445E)-Clamp ("open form") were extracted from **Figure 2** in the main text for comparison.

(**C**) WNT stimulation experiments with full-length (FL) FZD6-DEP-Clamp. Experiments were performed in HEK293A cells transiently transfected with the FZD6-DEP-Clamp sensor. Data show mean values ± SEM from three (for WNT-3A, WNT-5B and WNT-16B) to four (for WNT-5A) independent experiments.

Statistical significance was assessed using either two-tailed Student's t-test (in (**A**)) or one-way ANOVA followed by Tukey's post-hoc test (in (**B**)). Corresponding p-values are indicated in the respective figure panel.

Figure S4

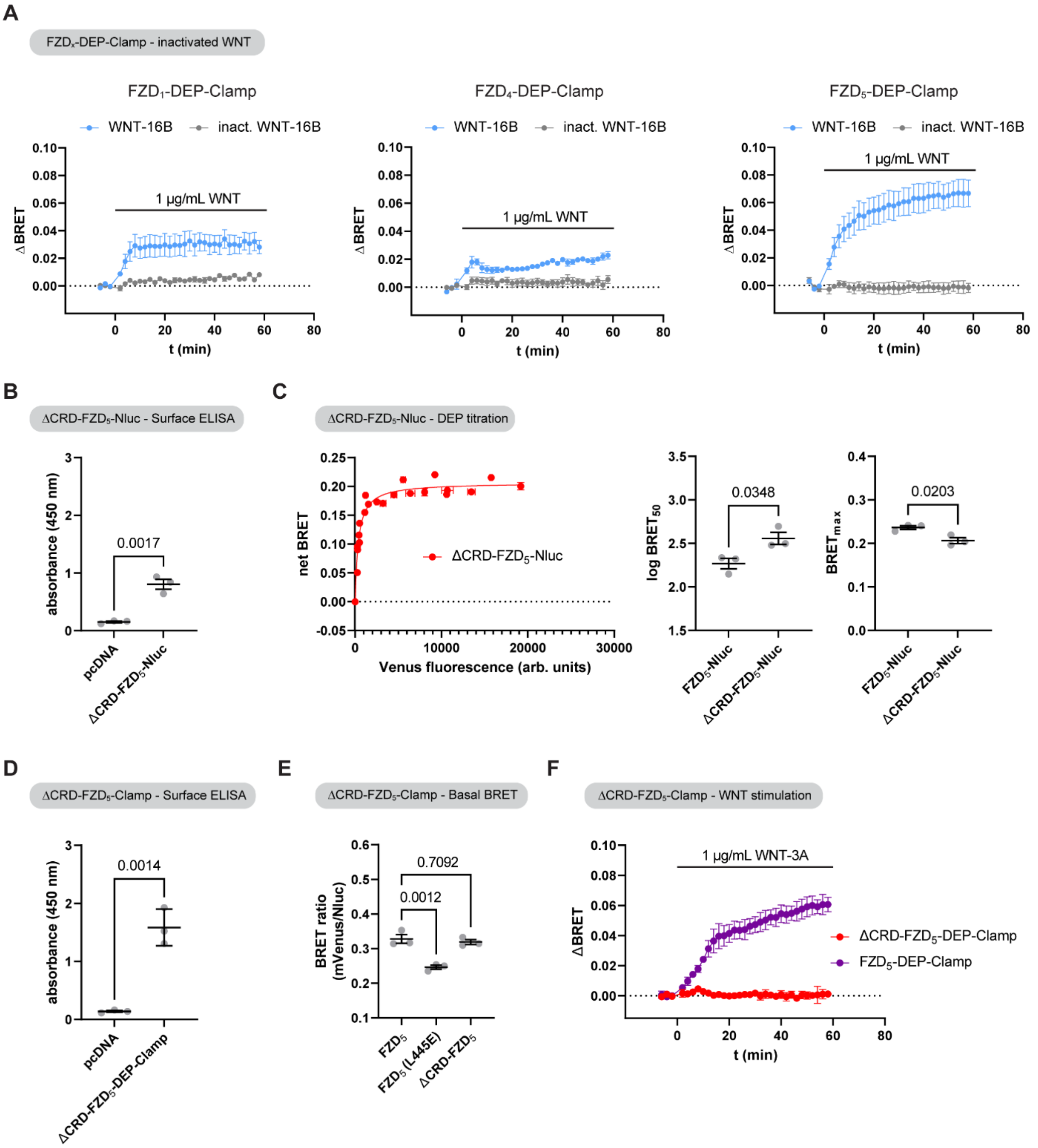
A
FZDx-DEP-Clamp - inactivated WNT
FZD1-DEP-Clamp
FZD4-DEP-Clamp
FZD5-DEP-Clamp
WNT-16B
inact. WNT-16B
1 μg/mL WNT
ΔBRET
t (min)
B
ΔCRD-FZD5-Nluc - Surface ELISA
absorbance (450 nm)
0.0017
pcDNA
ΔCRD-FZD5-Nluc
C
ΔCRD-FZD5-Nluc - DEP titration
net BRET
Venus fluorescence (arb. units)
ΔCRD-FZD5-Nluc
log BRET50
0.0348
BRETmax
0.0203
FZD5-Nluc
ΔCRD-FZD5-Nluc
D
ΔCRD-FZD5-Clamp - Surface ELISA
absorbance (450 nm)
0.0014
pcDNA
ΔCRD-FZD5-DEP-Clamp
E
ΔCRD-FZD5-Clamp - Basal BRET
BRET ratio (mVenus/Nluc)
0.7092
0.0012
FZD5
FZD5 (L445E)
ΔCRD-FZD5
F
ΔCRD-FZD5-Clamp - WNT stimulation
1 μg/mL WNT-3A
ΔBRET
t (min)
ΔCRD-FZD5-DEP-Clamp
FZD5-DEP-Clamp

Figure S4 (related to Figure 3): Proof of specificity for WNT-induced BRET responses seen for the FZD-DEP-Clamp sensors.

(**A**) Experiments with heat-inactivated WNT-16B. HEK293A cells were transiently transfected with the FZD_1-DEP-Clamp, the FZD_4-DEP-Clamp or the FZD_5-DEP-Clamp and stimulated with 1 µg/mL regular WNT-16B or heat-inactivated WNT-16B. Data show mean values ± SEM from three independent experiments performed in triplicate.

(**B**) Surface expression analysis of ΔCRD-FZD_5-Nluc and ΔCRD-FZD_5-DEP-Clamp. Plasma membrane expression was analyzed via surface ELISA using an antibody directed against the N-terminal HA tag. Experiments were performed in HEK293A cells transiently transfected with the indicated construct. Data show mean values ± SEM from three independent experiments performed in triplicate.

(**C**) DEP titration experiments with ΔCRD-FZD_5-Nluc and extracted parameters. Experiments were performed in HEK293A cells transiently transfected with a constant amount of ΔCRD-FZD_5-Nluc and increasing amounts of DEP-Venus. Titration data are shown as superimposed values ± SD (in x and y), while extracted parameters (log $BRET_{50}$ and $BRET_{max}$), which are derived from non-linear regression of each independent experiment, represent mean values ± SEM from three independent experiments performed in triplicate. Note that data for FZD_5-Nluc were copied from **Figure 1** in the main text for comparison purposes.

(**D**) Surface expression analysis of ΔCRD-FZD_5-DEP-Clamp. Plasma membrane expression was analyzed via surface ELISA using an antibody directed against the N-terminal HA tag. Experiments were performed in HEK293A cells transiently transfected with the indicated construct. Data show mean values ± SEM from three independent experiments performed in triplicate.

(**E**) Basal BRET ratio of ΔCRD-FZD_5-DEP-Clamp (in the absence of any ligand). Experiments were performed in HEK293A cells transiently transfected with the indicated constructs. Data show mean values ± SEM from three to four independent experiments performed in triplicate. Note that data for FZD_5-DEP-Clamp and FZD_5-DEP(L445E)-Clamp were extracted from **Figure 2** in the main text for comparison purposes.

(**F**) WNT-3A stimulation experiments with ΔCRD-FZD_5-DEP-Clamp in comparison with full-length FZD_5-DEP-Clamp. Experiments were performed in HEK293A cells transiently transfected with the indicated FZD-DEP-Clamp sensor. Data show mean values ± SEM from three independent experiments performed in triplicate.

Statistical significance was assessed using either two-tailed Student's t-test (in (**B**-**D**)) or one-way ANOVA followed by Dunnett's post-hoc test (in (**E**)). Corresponding p-values are indicated in the respective figure panel.

Figure S5

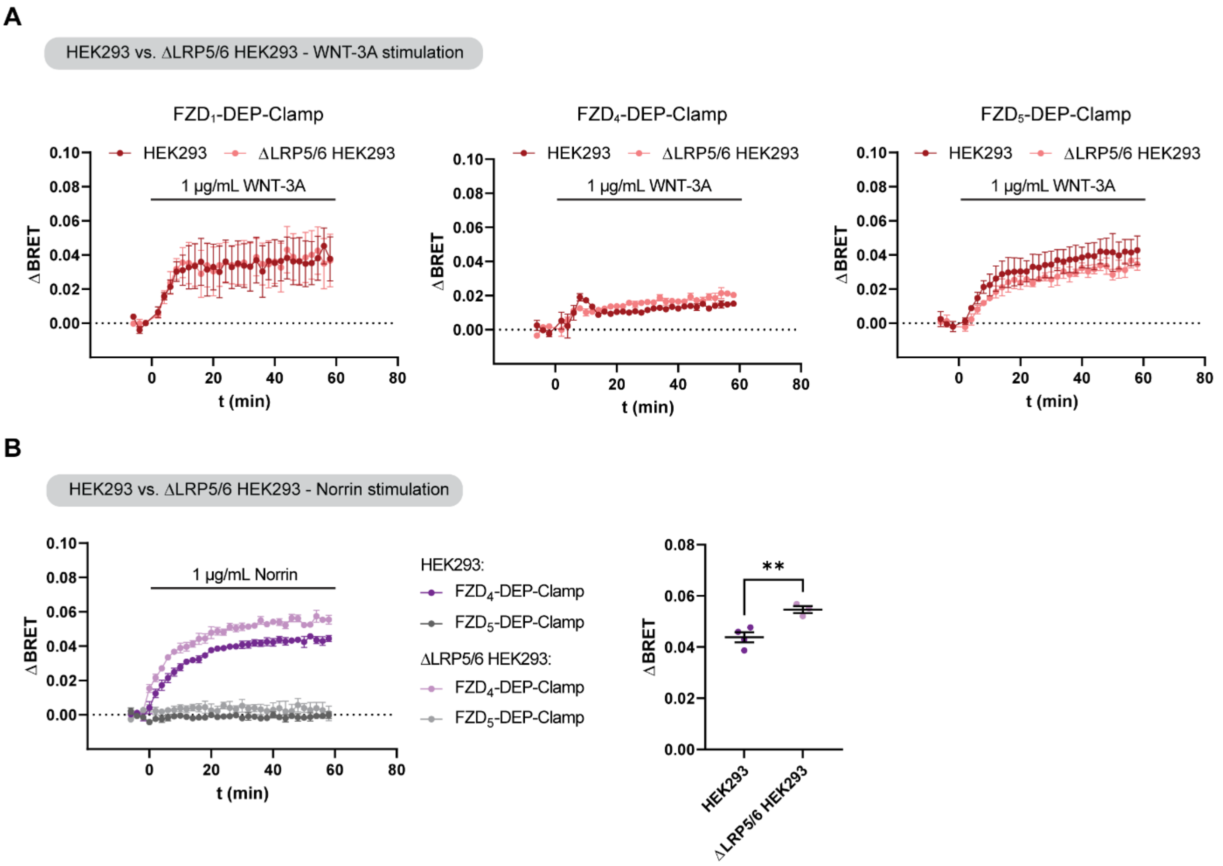

Figure S5: Impact of LRP5/6 on the WNT- or Norrin-induced BRET responses at selected FZD-DEP-Clamps.

(**A**) Investigation of LRP5/6-dependence of WNT-3A-induced FZD-DEP-Clamp responses. Experiments were performed in HEK293A or ΔLRP5/6 HEK293T cells transiently transfected with the FZD_1-, FZD_4- or FZD_5-DEP-Clamp. Data represent mean values ± SEM from three independent experiments (per cell line) performed in triplicate.

(**B**) Assessment of Norrin-induced FZD-DEP-Clamp responses (FZD_4-DEP-Clamp and FZD_5-DEP-Clamp) in the presence or absence of endogenous LRP5/6. Experiments were performed in HEK293A or ΔLRP5/6 HEK293T cells transiently transfected with the FZD_4- or FZD_5-DEP-Clamp. ΔBRET values (right panel) were extracted from experiments with the FZD_4-DEP-Clamp and represent the average of the last five measured values (from each independent experiment). Data represent mean values ± SEM from three to four independent experiments (per cell line) performed in triplicate. Statistical significance was assessed using Student's t-test (two-tailed). **: $p < 0.01$.

Figure S6

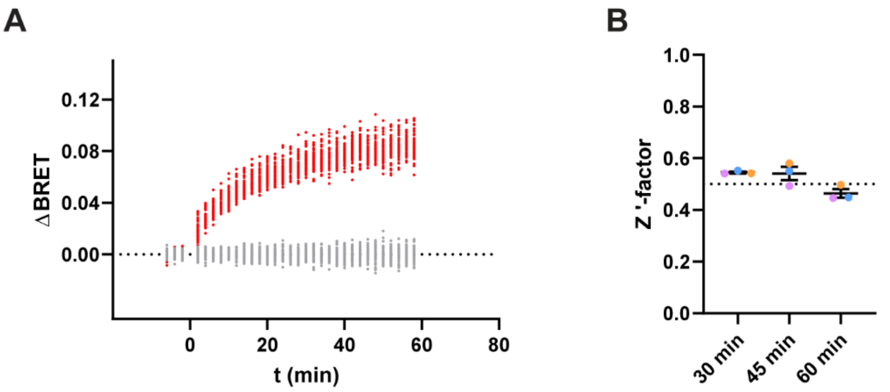

Figure S6 (related to Figure 5): Z' factor determination and time-dependence of the Z' factor for the FZD_5-DEP-Clamp.

(**A**) HEK293A cells stably expressing the FZD_5-DEP-Clamp were stimulated with 500 ng/mL WNT-3A (red symbols) or vehicle (grey symbols). Shown are the kinetic traces for technical replicates (n = 48) from one representative experiment.

(**B**) Time-dependence of the determined Z' factor. Different timepoints from the kinetic BRET responses were used to calculate the Z' factors. Data represent mean values ± SEM from three independent experiments. Each experiment consisted of 48 technical replicates.

Table S1 (related to Figure 1 and Figure S1): p-values from surface ELISA experiments with receptor-Nluc constructs.

p-values from Surface ELISA experiments with receptor-Nluc constructs. Experiments (*N* independent experiments, each in triplicate) were performed in HEK293A cells transiently transfected with the indicated receptor-Nluc construct.

Statistical significance against empty vector-transfected cells was assessed using one-way ANOVA (followed by Fisher's LSD post-hoc test). Significance levels were defined as follows: *: $p < 0.05$; **: $p < 0.01$; ***: $p < 0.001$; ****: $p < 0.0001$.

**receptor-Nluc**	**p-values (Surface ELISA, Figure S1)**	***N***
FZD_1-Nluc	0.0001; ***	4
FZD_2-Nluc	0.0008; ***	4
FZD_3-Nluc	0.0427; *	3
FZD_4-Nluc	< 0.0001; ****	3
FZD_5-Nluc	< 0.0001; ****	4
FZD_6-Nluc	< 0.0001; ****	4
FZD_6 Δ559-Nluc	0.0070; **	4
FZD_7-Nluc	< 0.0001; ****	4
$hFZD_8$-Nluc	0.0015; **	4
$mFZD_8$-Nluc	< 0.0001; ****	3
FZD_9-Nluc	< 0.0001; ****	4
FZD_{10}-Nluc	< 0.0001; ****	4
SMO-Nluc	< 0.0001; ****	4

Table S2 (related to Figure 2): p-values from surface ELISA experiments with FZD-DEP-Clamp constructs shown in Figure 2.

p-values from Surface ELISA experiments with different unimolecular FZD-DEP-sensors. Experiments were performed in HEK293A cells (*N* independent experiments performed in triplicate) transiently transfected with the indicated constructs.

Statistical significance against empty vector-transfected cells was assessed using one-way ANOVA (followed by Fisher's LSD post-hoc test). Significance levels were defined as follows: ns: not significant; **: $p < 0.01$; ****: $p < 0.0001$.

**FZD-DEP-sensor**	**p-values (Surface ELISA, Figure 2B)**	***N***
FZD_1-DEP (wild-type)	< 0.0001; ****	3
FZD_2-DEP (wild-type)	< 0.0001; ****	3
FZD_4-DEP (wild-type)	< 0.0001; ****	4
FZD_5-DEP (wild-type)	< 0.0001; ****	4
FZD_6 Δ559-DEP (wild-type)	0.0106; **	4
FZD_7-DEP (wild-type)	< 0.0001; ****	4
$mFZD_8$-DEP (wild-type)	< 0.0001; ****	4
FZD_9-DEP (wild-type)	< 0.0001; ****	3
FZD_{10}-DEP (wild-type)	< 0.0001; ****	4
FZD_1-DEP (L445E)	< 0.0001; ****	3
FZD_4-DEP (L445E)	< 0.0001; ****	3
FZD_5-DEP (L445E)	< 0.0001; ****	4
FZD_6 Δ559-DEP (L445E)	0.9328; ns	3

Table S3: Primers used in the study.

primer name	primer sequence (5' – 3')	Source
lin HA-FZD-XX_FW	GGCTCGAGTCTAGATGGAG	IDT DNA Technologies
lin HA-FZD-XX_RV	ATCCCGCATAATCCGGCAC	IDT DNA Technologies
Signal seq FZD_FW	CTGGCTAGTTAAGCTTCCACCATGCGGCTCTGCATCC	IDT DNA Technologies, Grätz and Kowalski-Jahn et al., 2023[1]
FZD_1+XbaI_RV	GATCTCTAGACTCGAGCCGACGGTGGTTTCCCCTTG	IDT DNA Technologies
FZD_7+XbaI_RV	GATCTCTAGACTCGAGCCTACCGCAGTCTCCCCCTTG	IDT DNA Technologies
$hFZD_8$+XbaI_RV	GATCTCTAGACTCGAGCCAACCTGACTCAATGGCATC	IDT DNA Technologies
FZD_2 for Cterm-Nluc_FW	GATGTGCCGGATTATGCGGGATCCTTCCACGGGGAGAAGGGCATC	IDT DNA Technologies
FZD_2 for Cterm-Nluc_RV	GCCACCTCCATCTAGACTCGAGCCCACGGTGGTCTCACCGTGTC	IDT DNA Technologies
FZD_3 for Cterm-Nluc_FW	TCCGTATGATGTGCCGGATTATGCGGGATCCCACAGTTTGTTTTC	IDT DNA Technologies
FZD_3 for Cterm-Nluc_RV	CCGCCACCTCCATCTAGACTCGAGCCAGCACTGGTTCCATCCTC	IDT DNA Technologies
ΔCRD FZD_5 G180_FW	ATGTGCCGGATTATGCGGGATCCGGAGAATGTCCTGCTGG	IDT DNA Technologies
FZD_5 for Cterm-Nluc_RV	GCCACCTCCATCTAGACTCGAGCCGACGTGGCTCAGAGACAC	IDT DNA Technologies
FZD_6 for Cterm-Nluc_FW	ATGTGCCGGATTATGCGGGATCCCACAGTCTCTTCACCTGTG	IDT DNA Technologies
FZD_6 for Cterm-Nluc_RV	GCCACCTCCATCTAGACTCGAGCCAGTATCTGAATGACAACCACCTC	IDT DNA Technologies
FZD_6 Δ559 for Cterm-Nluc_RV	GCCACCTCCATCTAGACTCGAGCCTCCCATGGATTTGGAAATGAC	IDT DNA Technologies
$mFZD_8$ for Cterm-Nluc_FW	GATGTGCCGGATTATGCGGGATCCGCTTCGGCCAAGGAGCTG	IDT DNA Technologies
$mFZD_8$ for Cterm-Nluc_RV	CCGCCACCTCCATCTAGACTCGAGCCGACCTGGGACAATGGCATTTG	IDT DNA Technologies

FZD_9 for Cterm-Nluc_FW	TGATGTGCCGGATTATGCGGGATCCCTGGAGATCGGCCGCTTC	IDT DNA Technologies
FZD_9 for Cterm-Nluc_RV	CACCTCCATCTAGACTCGAGCCGAGGTGTGTGGGGTTCTC	IDT DNA Technologies
SMO for Cterm-Nluc_FW	TCCGTATGATGTGCCGGATTATGCGGGATCCGCGGCCTTGAG	IDT DNA Technologies
SMO for Cterm-Nluc_RV	CCGCCACCTCCATCTAGACTCGAGCCGAAGTCCGAGTCTGCATC	IDT DNA Technologies
HA-FZD-Nluc into Clamp_FW	GTATGATGTGCCGGATTATGC	IDT DNA Technologies
HA-FZD-Nluc into Clamp_RV	CGCCACCTCCATCTAGAC	IDT DNA Technologies
lin pcDNA3.1 promoter_FW	TCTGGCTAACTAGAGAACCCAC	IDT DNA Technologies
lin pcDNA3.1 promoter_RV	GCGTATATCTGGCCCGTAC	IDT DNA Technologies
TK promoter for Gibson_FW	CGCGATGTACGGGCCAGATATACGCAAATGAGTCTTCGGACCTCG	IDT DNA Technologies
TK promoter for Gibson_RV	GCAGTGGGTTCTCTAGTTAGCCAGATTAAGCGGGTCGCTGCAG	IDT DNA Technologies

References

1. Grätz L, Kowalski-Jahn M, Scharf MM, Kozielewicz P, Jahn M, Bous J, Lambert NA, Gloriam DE, Schulte G. Pathway selectivity in Frizzleds is achieved by conserved micro-switches defining pathway-determining, active conformations. *Nat Commun*. 2023;14(1). doi:10.1038/s41467-023-40213-0

2. Kozielewicz P, Shekhani R, Moser S, Bowin CF, Wesslowski J, Davidson G, Schulte G. Quantitative Profiling of WNT-3A Binding to All Human Frizzled Paralogues in HEK293 Cells by NanoBiT/BRET Assessments. *ACS Pharmacol Transl Sci*. 2021;4(3):1235-1245. doi:10.1021/acsptsci.1c00084

3. Bowin CF, Kozielewicz P, Grätz L, Kowalski-Jahn M, Schihada H, Schulte G. WNT stimulation induces dynamic conformational changes in the Frizzled-Dishevelled interaction. *Sci Signal*. 2023;16(779). doi:10.1126/scisignal.abo4974

4. Zhang JH, Chung TDY, Oldenburg KR. A Simple Statistical Parameter for Use in Evaluation and Validation of High Throughput Screening Assays. *SLAS Discov*. 1999;4(2):67-73. doi: 10.1177/108705719900400206
